# Nested co-expression network analysis identifies compact gene clusters in a black box

**DOI:** 10.1093/bioinformatics/btag167

**Published:** 2026-04-03

**Authors:** I A Dyugay, A Poslavsky, D K Lukyanov, F M Polyakov, E Nikitin, E Klimuk, A Dakhnovets, D S Syrko, V V Kotliar, D M Chudakov

**Affiliations:** Center for Molecular and Cellular Biology, Moscow, Russia; Institute of Translational Medicine, Pirogov Russian National Research Medical University, Moscow, Russia; Genomics of Adaptive Immunity Department, Shemyakin and Ovchinnikov Institute of Bioorganic Chemistry, Moscow, Russia; Abu Dhabi Stem Cell Center, Al Muntazah, United Arab Emirates; Genomics of Adaptive Immunity Department, Shemyakin and Ovchinnikov Institute of Bioorganic Chemistry, Moscow, Russia; Center for Molecular and Cellular Biology, Moscow, Russia; Institute of Translational Medicine, Pirogov Russian National Research Medical University, Moscow, Russia; Genomics of Adaptive Immunity Department, Shemyakin and Ovchinnikov Institute of Bioorganic Chemistry, Moscow, Russia; Genomics of Adaptive Immunity Department, Shemyakin and Ovchinnikov Institute of Bioorganic Chemistry, Moscow, Russia; Biotech Campus LLC, Moscow, Russia; Biotech Campus LLC, Moscow, Russia; Center for Molecular and Cellular Biology, Moscow, Russia; Institute of Translational Medicine, Pirogov Russian National Research Medical University, Moscow, Russia; Genomics of Adaptive Immunity Department, Shemyakin and Ovchinnikov Institute of Bioorganic Chemistry, Moscow, Russia; Institute of Translational Medicine, Pirogov Russian National Research Medical University, Moscow, Russia; Genomics of Adaptive Immunity Department, Shemyakin and Ovchinnikov Institute of Bioorganic Chemistry, Moscow, Russia; Dmitry Mendeleev University, Moscow, Russia; Institute of Translational Medicine, Pirogov Russian National Research Medical University, Moscow, Russia; Center for Molecular and Cellular Biology, Moscow, Russia; Institute of Translational Medicine, Pirogov Russian National Research Medical University, Moscow, Russia; Genomics of Adaptive Immunity Department, Shemyakin and Ovchinnikov Institute of Bioorganic Chemistry, Moscow, Russia; Abu Dhabi Stem Cell Center, Al Muntazah, United Arab Emirates

## Abstract

**Motivation:**

Digital analysis of biological systems requires methods capable of identifying both broad and nested gene modules reflecting complex biological processes. Existing transcriptomic methods often miss compact gene sets corresponding to subprocesses in specialized cell types, limiting insights into functional heterogeneity.

**Results:**

We present Nested-WGCNA, a two-stage unsupervised network analysis algorithm designed to identify coarse-grained and fine-grained gene modules. Applied to bulk RNA-Seq data, Nested-WGCNA reveals stable modules reproducible across datasets. When validated against scRNA-Seq data, these modules correspond to both major and minor immune cell subtypes. Application to immunotherapy response datasets uncovers predictive and prognostic biomarkers, highlighting its utility in treatment stratification and biomarker discovery.

**Availability:**

The NestedWGCNA source code and analysis pipeline are available on GitHub (https://github.com/ilyada/NestedWGCNA) and archived on Zenodo (https://doi.org/10.5281/zenodo.18959244).

## 1 Introduction

Cancer treatment remains a hard challenge for the modern healthcare system. Tumor cells exploit a multitude of immunological tolerance mechanisms to escape immune response, which makes them difficult to eliminate (Bhatia and Kumar 2014). Immunotherapy has revolutionized cancer treatment by harnessing the immune system and directing cytotoxic cells to attack cancer cells (Ribas and Wolchok 2018). However, only a fraction of patients respond to immunotherapy, and existing predictive methods are relatively weak, calling for the identification of biomarkers to properly stratify patients (Sharma et al. 2017). To date, there are no biomarkers that could reliably predict response to anti-PD1/PD-L1 immune checkpoint therapy (Mariam et al. 2023). Diverse immunotherapeutic approaches are in development, exploiting different aspects of the tumor immune environment (Binnewies et al. 2018), where it may become even more challenging to predict response.

In the realm of biomarker discovery, the utilization of bulk transcriptomic data has gained prominence (Finotello et al. 2019, Bagaev et al. 2021). Such biomarkers, in particular, can be meaningful groups of genes that reflect certain biological processes or the presence of certain types of cells. Various approaches are used to identify such meaningful groups of genes (Sheng et al. 2020). Gene Regulatory Network is a concept of systems biology that aims to comprehensively model regulatory relationships between genes based on empirically accumulated information (Karlebach and Shamir 2008). In contrast, Gene Co-Expression Networks (GCNs) do not contain any information about causal relationships between genes, instead representing the transcript-transcript correlational relationships as undirected graphs in which nodes represent individual genes and edges represent co-expression association between genes. GCNs is a powerful concept aimed to extract biologically relevant information. It is employed to identify genes associated with certain phenotypes or to interpret molecular mechanisms of different biological processes (Ruan et al. 2010). In the last few years, GCNs have been used to find connections between groups of co-expressed genes (*gene modules*) and the development of different diseases (Xiang et al. 2018), the response to treatments (Xing et al. 2022).

The most widely used approach for GCN inferring and analysis is Weighted Gene Co-Expression Network Analysis, WGCNA (Langfelder and Horvath 2008). WGCNA can be applied to RNA-Seq data for the discovery of associations between certain phenotype and gene co-expression modules (Chen et al. 2019). However, this method is limited by issues such as noise, robustness, and scalability as exemplified below. The original WGCNA employs a square adjacency matrix based on pairwise gene correlations raised to an arbitrary coefficient beta to obtain a scale-free network. However, the rationale for scale-free transformation is debatable (Broido and Clauset 2019), and tuning the non-intuitive beta parameter is necessary.

Here we first re-worked WGCNA to achieve better robustness and reproducibility. One of the features of our modified WGCNA approach is that we use a fixed beta parameter–the parameter that is employed to raise the gene pair correlation value to the corresponding power (Langfelder and Horvath 2008). A fixed beta parameter allowed us to create the adjacency matrix from the Coefficients of determination, providing intuitive adjacency coefficients without the need for scale-free transformation. Additionally, we address issues with the dissimilarity measure in the original WGCNA, highlighting that it is the Szymkiewicz-Simpson coefficient which poses problems like non-zero dissimilarity for genes paired with itself and potential inaccuracies in connectivity-based configurations, leading to misclustering. The last problem is that there is no proof that dissimilarity measure is a metric. Importantly, the measure’s failure to be a metric means methods relying on triangle inequality won’t work because the necessary conditions for those methods, such as consistent distance relationships among points, are not met.

**Figure 1:**
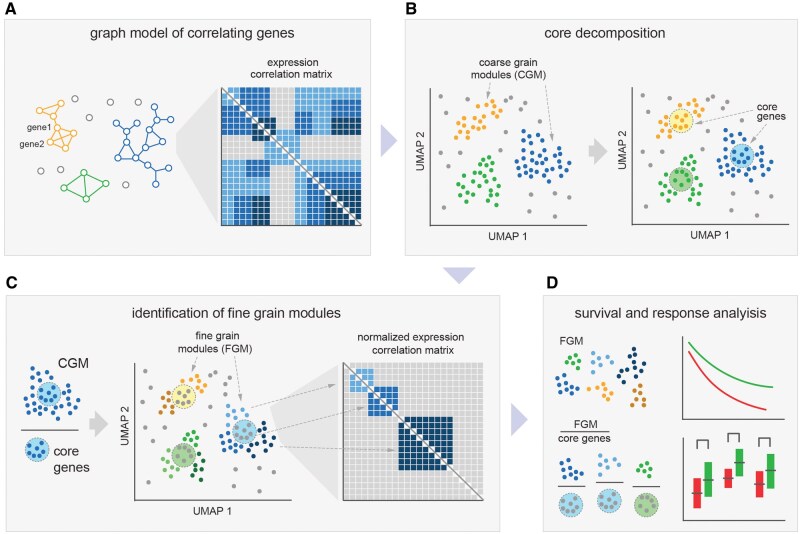
Overall scheme of Nested-WGCNA pipeline. Step-by-step methodology for constructing and analyzing gene co-expression networks to identify both coarse-grained and fine-grained gene modules is shown. (A) Gene co-expression network is initially built based on gene pair correlation values, followed by the calculation of adjacency and dissimilarity measures. (B) Using the HDBSCAN algorithm, coarse-grained modules (CGMs) of co-expressed genes are identified, and their cores are extracted using a core decomposition algorithm. (C) To find fine-grained modules (FGMs), all genes of each CGM are normalized by their core gene set and undergo repeated procedure of co-expression network inference. This results in smaller, correlated gene sets, with each FGM representing a subprocess within its parent CGM. (D) FGMs, or FGMs normalized on the core of their respective CGMs, are verified for association with the response and survival of cancer patients.

A major challenge in co-expression network analysis is identifying gene groups that represent specific subprocesses, as they are often embedded within larger gene modules associated with major biological processes. To date, no effective solution has been proposed to resolve this issue. To tackle this challenge, we developed a double-layer algorithm of GCN inference, named Nested-WGCNA (Fig. 1), allowing one to rationally divide identified gene coexpression modules to the more compact clusters.

At the first stage, we infer *Coarse-Grained Modules* (CGMs) on co-expression matrices, which represent large cell types or major functional processes. At the second stage, we apply a core decomposition algorithm to the inferred CGMs to identify a group of CGM *core genes*, a tight cluster of most correlated genes reflecting the large cell type or process as a whole.

For each CGM, genes are normalized by its core gene set and afterwards undergo repeated procedure of co-expression network inference. Such nested procedure yields more compact gene sets, named *Fine-Grained Modules* (FGMs). FGMs essentially represent groups of genes that remain correlated after being normalized on the major CGM component, which determines their self-standing nature. Following this logic, we suggest that each FGM reflects a subprocesses/cell subset within the large CGM. We next validate CGM cores and FGMs obtained from the bladder cancer and lung adenocarcinoma bulk transcriptomic data by mapping them on scRNA-Seq of lung adenocarcinoma environment (Leader et al. 2021).

Finally, we demonstrate that the resulting FGMs may be further explored as potential biomarkers for the survival and response to immunotherapy, whether unnormalized (abundance of a subprocess activity) or normalized against the corresponding *core genes* (proportion of a subprocess activity of a whole process). Cytotoxic gene signature, reflecting active CD8^+^ CD4^+^ CTL, and NK cell infiltration, expression of TGFB1, and IgG/IgA genes proportion have been reported to be correlated with response to atezolizumab (anti-PD-L1) in bladder cancer (Mariathasan et al. 2018, Dyugay et al. 2022). With Nested-WGCNA, this approach becomes applicable to any undisclosed process in a black-box manner. Here we show that FGM normalization on CGM core, similar in its concept to Immfocus normalization (Teltsh et al. 2015), enables informative estimations, such as determining the abundance of cytotoxic cells (Dyugay et al. 2022) or myeloid derived suppressor cells (Kumar et al. 2016) among all tumor-infiltrating immune cells, allowing to efficiently predict response to cancer immunotherapy.

## 2 Methods

### 2.1 Algorithm description

The original WGCNA consists of several stages: adjacency matrix creation from genes expressions; conversion of the adjacency matrix to dissimilarity matrix; module identification using values from dissimilarity matrix as distances; computation of eigengenes from modules. All these stages were improved in our work.

#### 2.1.1 Adjacency matrix

In the original WGCNA the adjacency matrix is a square matrix that consists of pairwise correlations between all genes in power of an arbitrary coefficient beta:


$$a_{ij}=\mathrm{corr}{\left(\mathrm{gen}e_{i},\mathrm{gen}e_{j}\right)}^{\beta }$$

The beta coefficient was initially introduced to make the resulting network scale-free. As discussed in the main text, bringing the network to a scale-free form may not have such a strong argumentation. Also, this parameter is not very intuitive and should be tuned. Here, we fixed the beta parameter to make the adjacency matrix consist of Coefficient of determination:


$$a_{ij}=\mathrm{corr}{\left(\mathrm{gen}e_{i},\mathrm{gen}e_{j}\right)}^{2}$$

Сoefficient of determination brings strong intuition to adjacency coefficients.

#### 2.1.2 Dissimilarity matrix

The dissimilarity measure used in the original WGCNA framework, was calculated by the following formula:


$$\mathrm{dissim}\left(\mathrm{gen}e_{i},\mathrm{gen}e_{j}\right)=1-\frac{\sum_{u=1}^{n}a_{iu}*a_{uj}+a_{ij}}{\min\left(\sum_{u=1}^{n}a_{iu},\sum_{u=1}^{n}a_{ju}\right)+1-a_{ij}}$$

The authors refer to the right part of the formula as the Topological Overlap Measure. But actually, it’s the Szymkiewicz-Simpson coefficient. For two genes *i* and *j*, this coefficient is defined as:


$$S\left(i,j\right)=\frac{\left|N\left(i\right)\cap N\left(j\right)\right|}{\min\left(\left|N\left(i\right)\right|,\left|N\left(j\right)\right|\right)}$$

where *N* (*i*) and *N* (*j*) are the neighboring sets of genes *i* and *j*. Intuitively, this coefficient measures the fraction of the smaller neighborhood that is shared between the two nodes. While this definition captures overlap, it has several drawbacks when applied to gene co-expression networks.

A significant pitfall in gene co-expression network analysis, such as with WGCNA, concerns the handling and behavior of housekeeping genes. Housekeeping or hub genes, which are fundamental to basic cellular functions and expressed across nearly all cell types, typically show broad co-expression patterns with a large spectrum of other genes. In WGCNA networks, this widespread co-expression causes housekeeping genes to frequently emerge as network hubs or be assigned to multiple modules simultaneously. Such broad connectivity blurs the boundaries between distinct modules and diminishes their biological specificity, making it challenging to associate modules distinctly with particular cellular processes (van Dam et al. 2017).

This phenomenon can be traced in part to properties of the Szymkiewicz-Simpson coefficient, used to measure pairwise dissimilarity between gene connectivity profiles in WGCNA. Notably, the coefficient is calculated as the size of the intersection of two sets divided by the minimum size of either set. One consequence is that if all neighbors of gene A are also neighbors of gene B, even if gene B has many more neighbors not shared by A, the dissimilarity between A and B is considered zero. This configuration makes it impossible to distinguish between completely overlapping and “core-periphery” relationships in the network. In practice, this is precisely the scenario for housekeeping genes, which often share many connections with pathway-specific genes: the overlap coefficient fails to differentiate between a pair of genes specifically co-expressed and one gene that simply connects to almost everything. This can distort the modular topology of networks, yielding a “core-periphery” structure dominated by central (core) genes, including housekeeping genes, which artificially link otherwise unrelated modules (Melo et al. 2024).

Furthermore, methodological issues compound the challenge. First, by this formula, a gene compared with itself is not strictly assigned a similarity of one. In the original R implementation, the authors masked this by hard-coding zero dissimilarity on the main diagonal, but if you duplicate genes in your network, these copies are not perfectly similar, exposing inconsistencies. Second, because of minimum connectivity in the denominator of the coefficient, some configurations may yield misleading values. For example, if the connection between two genes is 1, and all neighbors of the first gene are common with the second, but the second gene has many other neighbors, the dissimilarity between them would be zero. Same as if all neighbors of both genes would be exclusively common (Fig. S3). It makes these two different situations indistinguishable and is a primary reason why WGCNA mistakenly includes housekeeping genes, which likely have more connections with other genes than an average gene, within clusters. Moreover, since the overlap coefficient is not a true metric, it does not satisfy all properties such as the triangle inequality, analytic methods relying on metric properties may yield invalid results or interpretations.

As highlighted in methodological reviews and simulation studies (van Dam et al. 2017, Melo et al. 2024), these characteristics can distort module definition, reduce biological interpretability, and create “hub” artifacts in the output of WGCNA. Therefore, careful consideration of housekeeping gene properties, overlap coefficient limitations, and their combinatorial effects is essential for avoiding misleading conclusions in gene co-expression analysis. Our approach seeks to mitigate these artifacts, for example by separating broadly connected housekeeping genes from functionally coherent clusters, thereby preserving the specificity and biological relevance of detected modules.

During our work many other similarity measures were tested (including Jaccard, Ruzicka, etc.), but all of them were breaking the topology of the initial space (data not shown). Best results were yielded by the following measure:


$$\mathrm{dissim}\left(\mathrm{gen}e_{i},\mathrm{gen}e_{j}\right)=\sqrt{1-a_{ij}}$$

which, combined with Coefficient of determination as adjacency value, makes the dissimilarity a metric (Chen et al. 2023).

#### 2.1.3 Module identification

The average linkage hierarchical clustering of genes with Dynamic Tree Cut heuristic is replaced by the state-of-the-art HDBSCAN clustering algorithm. But, as already stated, the HDBSCAN is vulnerable to the “curse of dimensionality” and yields poor results on datasets with roughly more than one hundred dimensions (McInnes et al. 2017) To address this problem, we used the UMAP dimensionality reduction tool prior to clustering. For the end user, the only hyperparameter necessary to choose is a very intuitive minimum cluster size.

We dynamically determine the number of UMAP components based on the dataset size, setting it to approximately **log⁡2(N)+1**, where **N** is the number of samples. This heuristic is motivated by the idea that as the dataset size increases, the intrinsic dimensionality of the data also tends to increase, necessitating a greater number of components to capture relevant variance. A fixed low-dimensional representation (2D or 3D) might result in excessive information loss, while too many dimensions could introduce noise. The logarithmic scaling provides a balance between computational efficiency and information retention.

The choice of **n_neighbors** in UMAP plays a critical role in balancing local and global structure preservation. We set n_neighbors = 2 * min_clust_size to ensure well-connected clusters and reduce the risk of fragmentation that could negatively impact clustering stability. A too-small **n_neighbors** could lead to over-segmentation of clusters, while an excessively large value could smooth out meaningful structures. Our choice helps maintain dense clusters while capturing broader relationships between data points.

HDBSCAN provides multiple tunable parameters beyond the minimum cluster size. While our method primarily relies on **min_cluster_size** as a biologically interpretable parameter, other arguments like **min_samples** can influence the final clustering. However, our approach follows the practice of fixing **min_samples** to half of **min_cluster_size**.

For clarity, we provide a pseudocode overview of the Nested-WGCNA algorithm below:

Algorithm 1 CGM Identification

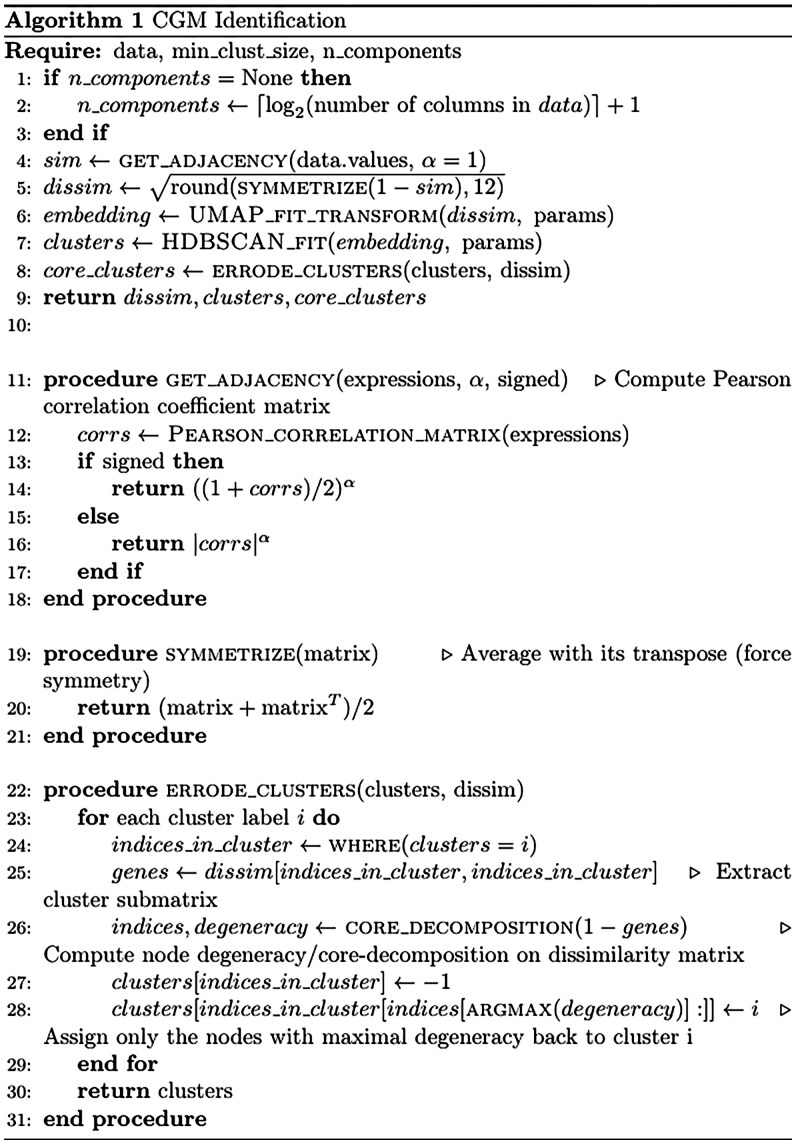

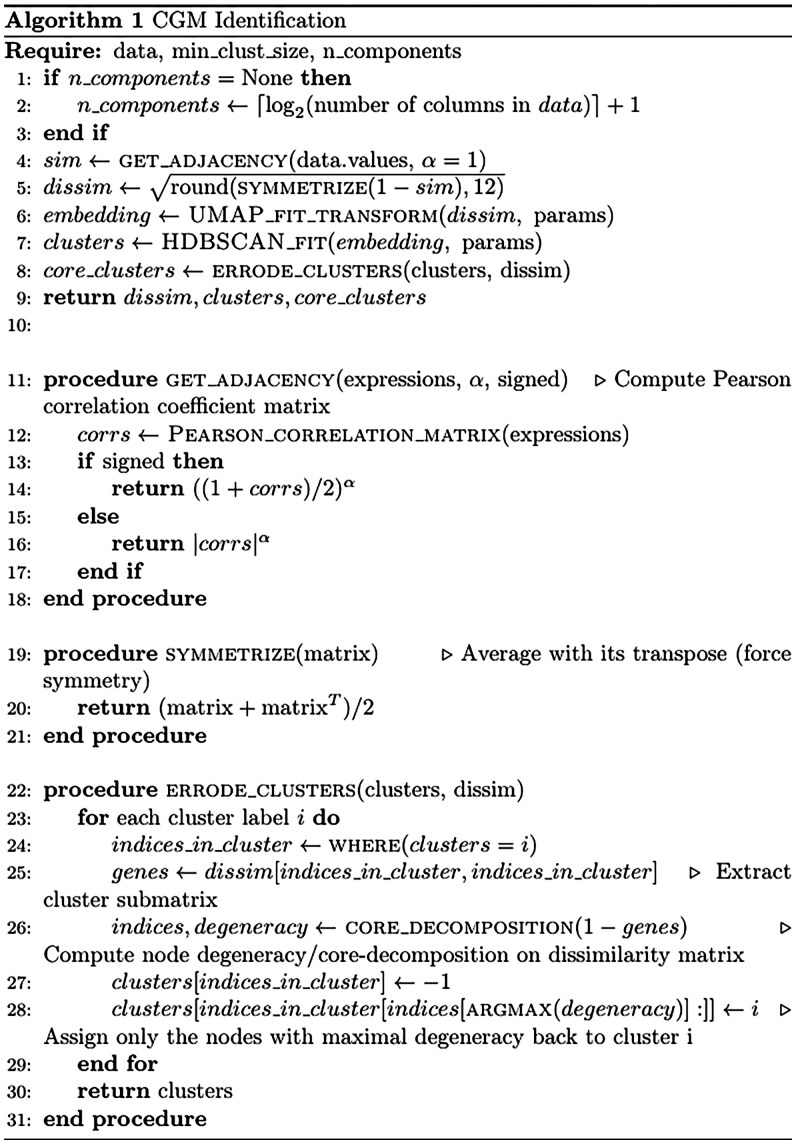


#### 2.1.4 Core decomposition

The core decomposition of an unweighted graph assigns an integer (the core number) to each node, indicating its level of connectivity with its neighbors. Nodes are iteratively removed, starting with the least connected ones, and the core number is assigned as the highest value *k* for which they remain in the *k*-core. This process continues until all nodes are assigned their core numbers, revealing the subgraph structure based on connectivity. The definition of a core of a weighted graph, however, is slightly different. The *k*-core of a weighted graph G is the maximal induced subgraph G_k_ where for every node u in G_k_, the sum of the weights of the edges connecting u to other nodes within Gk is at least *k*. The core number *c (u)* of a node *u* is the highest value of *k* such that *u* belongs to the *k*-core. Core decomposition algorithm is applied to clusters discovered in dissimilarity matrices (Malliaros et al. 2020).

This concept is strongly related to the concept of graph degeneracy. The graph degeneracy is the highest core number in the graph. It means that there is no subgraph in this graph that has higher connectivity of the most loosely connected node.

Core decomposition allows us to find the most dense parts of the graph, like maximum clique or complete bipartite subgraph. Presumably, the maximum core may capture a central subset of elements that represent key players in the entire environmental system.

For clarity, we provide a pseudocode overview of the Core Decomposition algorithm below:


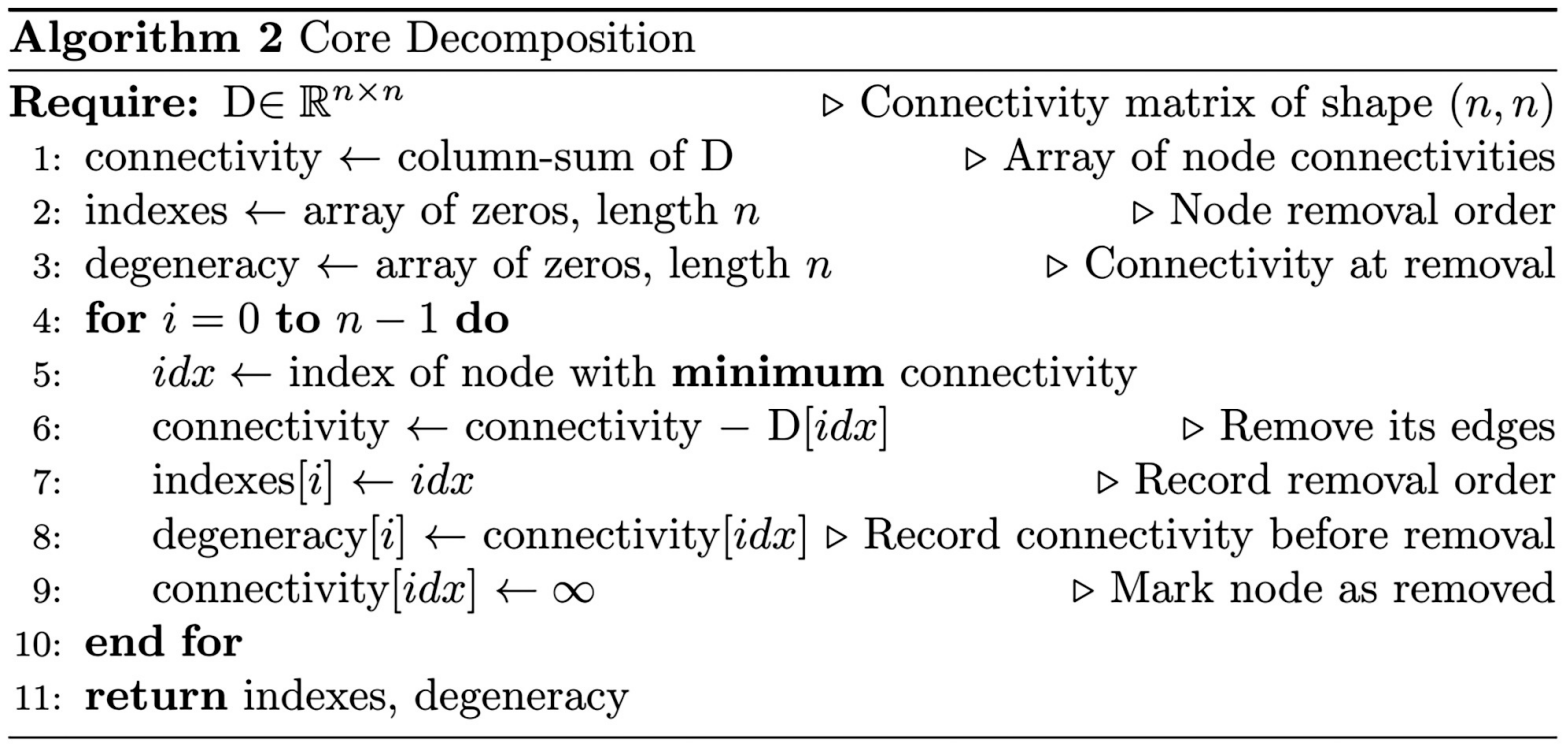

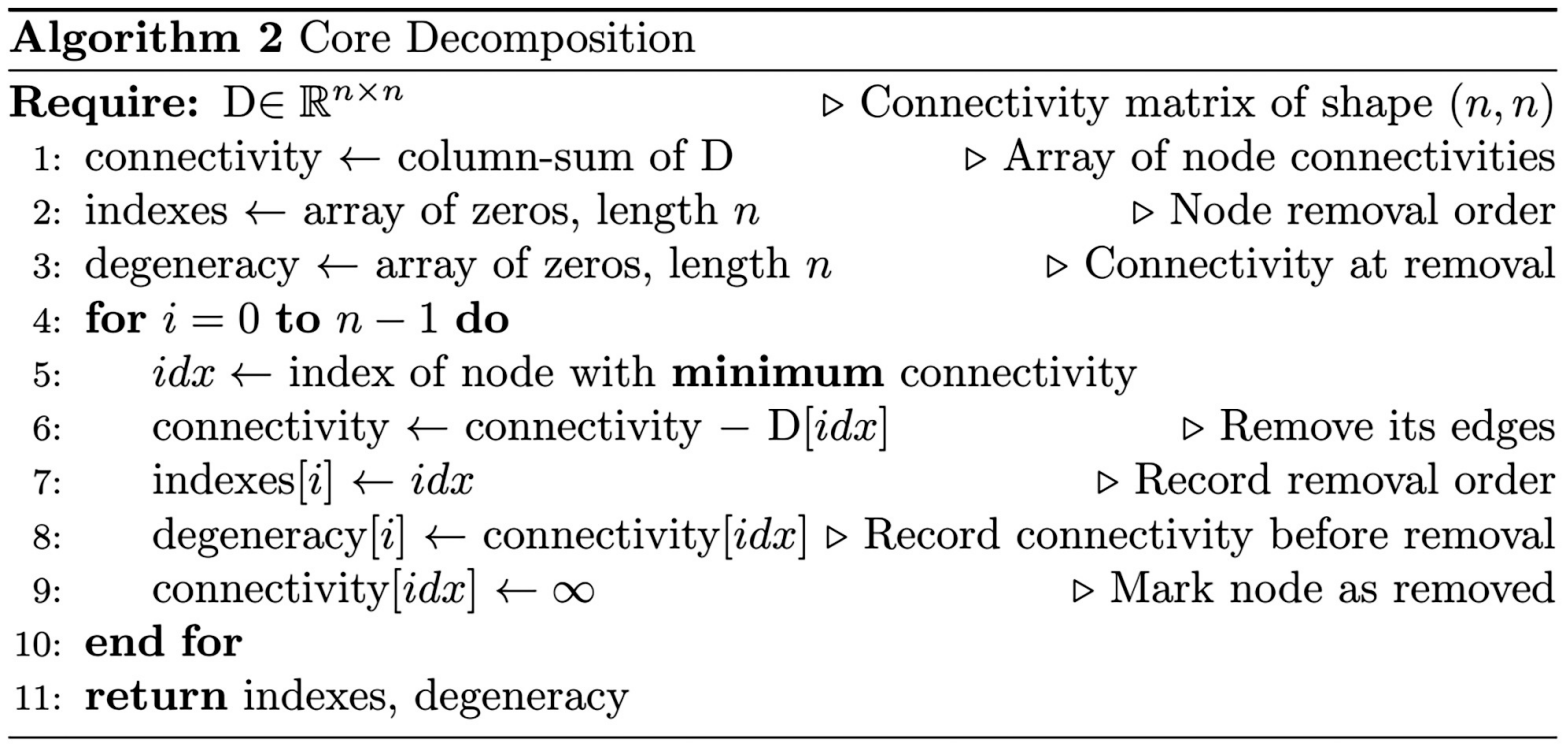

### 2.2 Dataset description

#### 2.2.1 Bulk RNA-Seq data

In this study, we utilized patient RNA-Seq data from TCGA-BLCA (Weinstein et al. 2014) with 406 cases, TCGA-KIRC (Creighton et al. 2013) with 533 cases, and TCGA-LUAD (Collisson et al. 2014) with 517 cases. Patient data from the ImVigor210 clinical trial (NCT02108652) and ImMotion150 clinical trial (NCT01984242) were obtained from the European Genome-Phenome Archive. The ImVigor210 clinical trial included participants with locally advanced or metastatic urothelial bladder cancer who were either treatment-naïve and ineligible for cisplatin-containing chemotherapy (*n* = 119) or had progressed during or after a prior platinum-based chemotherapy regimen (*n* = 310). This trial included RNA-seq tissue transcriptomic data for 345 patients. The ImMotion150 clinical trial was designed to evaluate the safety and provide preliminary evidence of the activity of atezolizumab + bevacizumab versus sunitinib, as well as atezolizumab monotherapy versus sunitinib. Each arm had a sample size of approximately 100 patients. The data were transformed into transcripts per million (TPM).

Genes from each dataset were filtered by several criteria. First of all, noncoding genes were omitted. After that not only genes with constant expression, but also genes with Misclassification error (one minus maximum fraction of constant values) less than 1/e at least in one dataset were dropped. That was done due to the low amount of information in those genes and usage of bootstrap technique in following procedures, which generates pseudo samples that contain 1 − 1/e (≈0.632) of the data sample (Efron and Tibshirani 1997).

#### 2.2.2 scRNA-Seq data

LUAD. Processed publicly available 3′ scRNA-seq data containing UMI counts of 336002 total lung adenocarcinoma cells from donors was downloaded from https://github.com/effiken/Leader_et_al and uploaded to Seurat R package (Butler et al. 2018) (v4.2.0). The downstream analysis included standard Seurat normalization, identification of variable genes, running PCA, Louvain clustering and UMAP algorithm with 20 dimensions based on ElbowPlot function result. All IG and TCR genes were excluded from the variable features utilized in the PCA. In the dataset, 54% of cells were collected from tumor sites and 46% originated from normal lung tissue. CGMs and FGMs were calculated as signature scores for each cell using the AddModuleScore Seurat function with default parameters. The annotation provided by the authors of the dataset was used to define cell lineages.

Atherosclerosis. The atherosclerotic dataset used in our study was built by integrating data from three studies (Fernandez et al. 2019, Chowdhury et al. 2022, Gayoso et al. 2022). The data from all studies were downloaded from NCBI GEO (GEO accession numbers: GSE196943, GSE196943, and GSE210152).

Cells from the Fernandez et al. (2019) dataset containing fewer than 200 counts, more than 4000 genes, or more than 7% mitochondrial genes were marked as outliers and removed. Cells from the Chowdhury et al. (2022) dataset containing fewer than 500 genes or more than 5% mitochondrial genes were marked as outliers and removed. Cells from the Gayoso et al. (2022) dataset containing fewer than 500 genes, more than 20 000 counts, or more than 10% mitochondrial genes were marked as outliers and removed.

scDblFinder was used to detect doublets, which were subsequently filtered out. scran normalization was used with subsequent log (1+p) transformation on the merged dataset. The top 2000 highly variable genes were identified using scanpy highly_variable_genes function with cell_ranger flavor.

The datasets were integrated using scVI, with “batch” and “article as categorical covariates,” and percentage of mitochondrial and ribosomal genes as continuous covariates. To determine the optimal clustering resolution, we employed nested Leiden clustering, as implemented in scanpy. The approach consisted of starting from small resolution values, followed by iterative reclustering of the resulting clusters. The process was repeated until the optimal resolution was achieved.

Finally, we performed manual annotation of clusters based on widely used sets of marker genes.

COVID-19. To showcase the applicability of our method on COVID-19 data, the dataset from Stephenson et al. (2021) was used. The dataset was downloaded from cellxgene portal and loaded into seurat environment. Prior to applying our algorithm, all cells belonging to healthy controls and respiratory system disorder patients were removed, yielding a dataset of 527286 cells from COVID-19 patients (Stephenson et al. 2021).

### 2.3 Imfocus-like normalization

Normalization procedure used in the current work was conducted similarly to Teltsh et al. (2015), with modifications. First, for selected coarse-grained modules (CGM) its core is obtained via core decomposition algorithm, and the corresponding eigengene is calculated as the first principal component. Second, the immune-normalized gene set (INGS), a group of genes with a Spearman correlation coefficient with corresponding eigengene >0.9 is selected. Next, the sample-specific normalization factor (fINGS) was calculated for each sample as the averaged expression of genes from INGS, and then the first normalization was performed. The normalization coefficient for genes included in INGS avoided self-normalization and was calculated as the averaged expression of the remaining genes. We selected genes from INGS for which the ratio between the coefficient of variation after and before the first normalization was <0.8 and used those genes as the final INGS. The second normalization was performed using the final INGS.

### 2.4 Gene module stability analysis

To evaluate reproducibility of identified modules, we employed three standard clustering agreement measures: Adjusted Rand Index (ARI), Adjusted Mutual Information (AMI), and the V-measure.

- *Bootstrap analysis*: From the IMvigor210 dataset, we generated 10 bootstrap pseudosamples, each containing approximately 63% of the original samples. For each pseudosample, CGMs were inferred using our Nested-WGCNA pipeline. Pairwise comparisons of clustering assignments across bootstrap replicates were then performed using ARI, AMI, and V-measure. The resulting scores were averaged across all pairs, and their standard deviations were computed to quantify stability.
- *Cross-dataset stability*: To assess module reproducibility across independent datasets of the same cancer type (e.g. bladder cancer), we applied the same pipeline to both datasets (IMvigor210 and TCGA BLCA). Modules derived from each dataset were compared pairwise using ARI, AMI, and V-measure to evaluate structural consistency of the clustering solutions.

These procedures provide a quantitative measure of module robustness both within a dataset (via bootstrap) and across datasets (via independent replication).

### 2.5 Enrichment analysis

In addition, to confirm that the identified core modules capture biologically meaningful processes, we performed enrichment analysis using. For each core module, we tested overrepresentation of xCell signatures using gseapy tool. Significant enrichments (adjusted *P* < 0.05) were used to annotate modules and verify correspondence with known biological functions.

### 2.6 Knowledge score computation

Knowledge scores were computed according to the procedure described in a recent article (Abbassi-Daloii et al. 2020). We used the ChEA_2022 database (Keenan et al. 2019) as a reference to retrieve experimentally validated transcription factor (TF)–target gene relationships derived from human ChIP-seq studies. Genes lacking TF annotation in ChEA_2022 were excluded from the analysis. To assess transcriptional coherence within CGMs and FGMs, we compared the observed co-expressed gene pairs (CPs) with knowledge pairs (KPs), and computed the knowledge score based on this comparison. In our research, knowledge pairs are defined as gene pairs that share at least one common TF regulator, while co-expressed pairs are defined as pairs of genes from the same gene module. The total number of gene pairs is calculated using the formula N × (N − 1)/2, where N represents the total number of TF-annotated genes in a particular gene set. Each gene pair is then categorized and counted into four groups: CP∩KP (pairs that are both CPs and KPs), ¬CP∩KP (pairs that are only KPs), CP∩¬KP (pairs that are only CPs), and ¬CP∩¬KP (pairs that are neither CPs nor KPs). The knowledge score is computed as follows: (CP∩KP × ¬CP∩¬KP)/(¬CP∩KP × CP∩¬KP). Statistical significance for the score was assessed using Fisher’s exact test. We utilized the knowledge score to evaluate the enrichment of transcriptionally co-regulated gene pairs within our CGMs and FGMs.

### 2.7 Statistical analysis

Survival analysis was performed with the lifelines (Davidson-Pilon 2024) Python library. Survival plots were created using the Kaplan–Meier estimator. Significance of the difference between two survival curves was estimated with a log-rank test. The area under the receiver operating curve (ROC AUC) was calculated with the Python scikit-learn library. For multiple comparisons, correction was performed using the Benjamini–Hochberg procedure (Benjamini and Hochberg 1995). Group comparison in boxplots was performed with the Kruskal–Wallis test. All statistical calculations were performed using Python. *P* < 0.05 was considered statistically significant.

### 2.8 Data availability

This study utilizes publicly available transcriptomic datasets. Bulk RNA-Seq data from TCGA, including BLCA, LUAD, and KIRC cancer types, were obtained from the Cancer Genome Atlas (TCGA) https://www.cancer.gov/tcga. Patient data from the ImVigor210 clinical trial (NCT02108652) and ImMotion150 clinical trial (NCT01984242) were obtained from the European Genome-Phenome Archive, subject to controlled access.

For single-cell RNA sequencing (scRNA-Seq) analyses: the LUAD dataset was downloaded from publicly available data (https://github.com/effiken/Leader_et_al). The atherosclerosis dataset was constructed by integrating data from three studies available in NCBI GEO (GEO accession numbers: GSE196943, GSE196943, and GSE210152). The COVID-19 dataset was obtained from the CellxGene portal.

### 2.9 Code availability

The NestedWGCNA source code and analysis pipeline are available on GitHub (https://github.com/ilyada/NestedWGCNA) and archived on Zenodo (https://doi.org/10.5281/zenodo.18959244).

## 3 Results

### 3.1 Identification of coarse-grained modules

First, we aimed to develop an optimal black-box algorithm to identify *Coarse-Grained Modules* (CGM) of correlated genes associated with major cell types and processes. The original WGCNA consists of several stages: *adjacency* matrix creation from gene expressions; conversion of the adjacency matrix to *dissimilarity* matrix; *module* identification using values from dissimilarity matrix as distances; *computation* of eigengenes from modules. Here we worked to optimize the first three stages as described in Methods. Application of this optimized approach to transcriptomic data from the IMVigor210 phase II clinical trial of atezolizumab (anti–PD-L1) in muscle-invasive urothelial carcinoma (Hoffman-Censits et al. 2016) yielded 28 CGM, each including from 93 to 957 genes. Nine thousand five hundred seventy-six genes remained unspecified (Supplementary Data 1–3).

### 3.2 Identification of coarse-grained module cores

**Figure 2:**
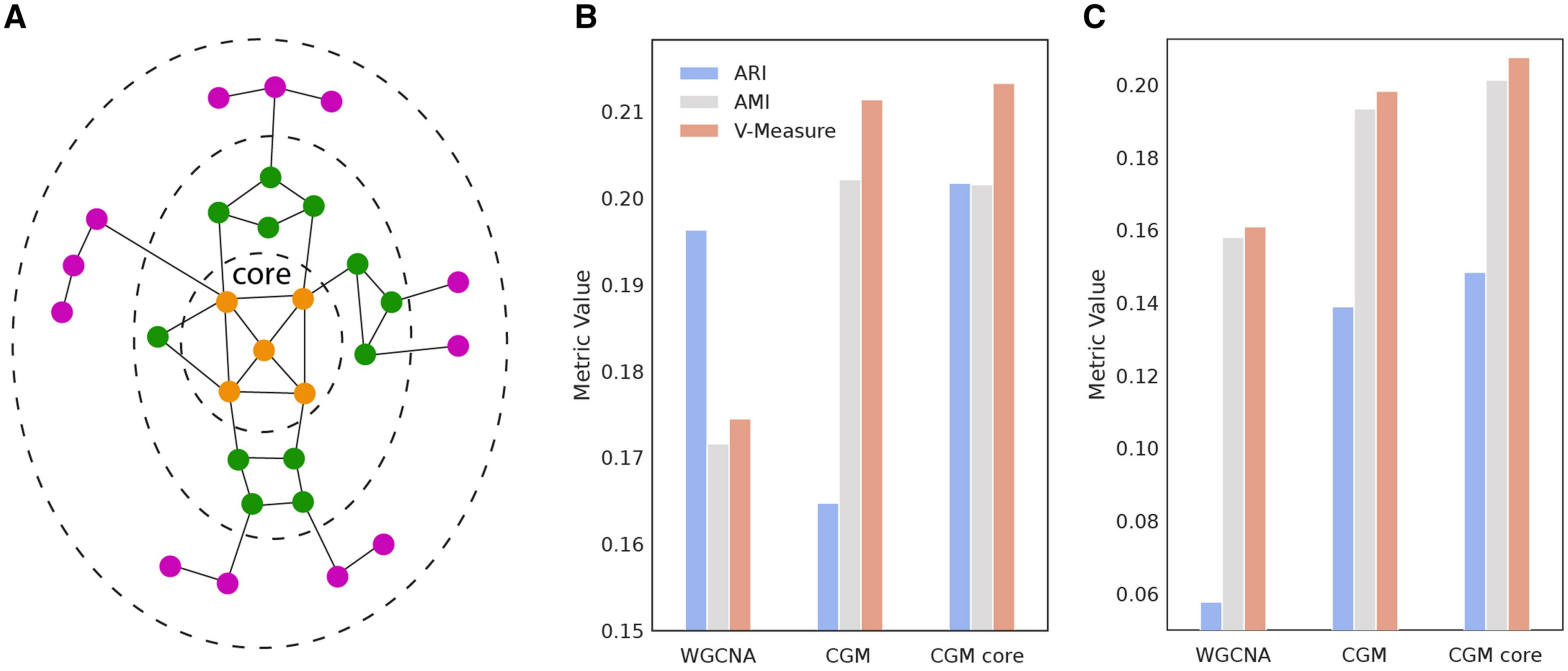
Core decomposition, reproducibility of gene clusters. (A) Principle of core decomposition. Adapted from Malliaros *et al*. (2020). (B and C) Reproducibility of gene clusters for bladder cancer RNA-Seq data (TCGA BLCA versus IMVigor210, b), and for renal cell carcinoma (TCGA KIRC versus ImMotion150, C). Adjusted Rand Index (ARI), Adjusted Mutual Information (AMI) and V-measure index values are shown for WGCNA, CGM, and CGM core gene clusters.

Next, in order to obtain a set of genes that comprehensively and stably describe the central functional process of each CGM, we applied the core decomposition approach (Malliaros et al. 2020). The core decomposition of an unweighted graph assigns an integer (the core number) to each node, indicating its level of connectivity with its neighbors. Nodes are iteratively removed starting with the least connected ones. The procedure stops when reaching the subgraph with the highest value of minimal connectivity [Fig. 2a, adapted from Malliaros et al. (2020)]. This procedure yielded CGM cores, each consisting of 30–70% of original CGM genes.

To estimate the algorithm’s reproducibility, we calculated corresponding metrics on the resulting gene clusters on different datasets related to the same cancer type. In particular, for bladder cancer we took TCGA BLCA and IMVigor210 (Hoffman-Censits et al. 2016), and for renal carcinoma we took TCGA KIRC and ImMotion150 (Atkins et al. 2017). This analysis revealed better performance of CGM cores compared to CGM and WGCNA gene groups (Fig. 2b and c). We also performed bootstrap analysis on 10 pseudosamples from the TCGA BLCA dataset (Weinstein et al. 2014) with default parameters of both algorithms. Again, CGM cores yielded better average metrics and lesser variance in comparison with WGCNA (Table 1).

**Table 1:** Bootstrap evaluation of algorithms performed on TCGA BLCA dataset.

	mARI	Cv (ARI)	mAMI	Cv (AMI)
WGCNA	0.21	23%	0.42	9%
CGM cores	0.42	9%	0.47	6%

mARI, mean Adjusted Rand Index; mAMI, mean Adjusted Mutual Information Cv (ARI), Coefficient of variation. The mARI and mAMI values represent the mean Adjusted Rand Index and Adjusted Mutual Information, respectively, and are used to assess clustering consistency across different bootstrap samples. In our analysis, these metrics were used to quantify the agreement between clustering solutions. The differences observed in these metrics are meaningful in the sense that higher values indicate more reliable and consistent clustering, and lower values indicate greater variability between bootstrap samples.

Next, we used the Enrichr tool (Xie et al. 2021) to evaluate biological relevance of obtained CGM cores. Each CGM core was annotated via hypergeometric test utilizing signatures sourced from the xCell tool (Aran et al. 2017) and signatures characterizing tumor environment from Bagaev et al. (2021) (Supplementary Data 1 and 2).

**Figure 3:**
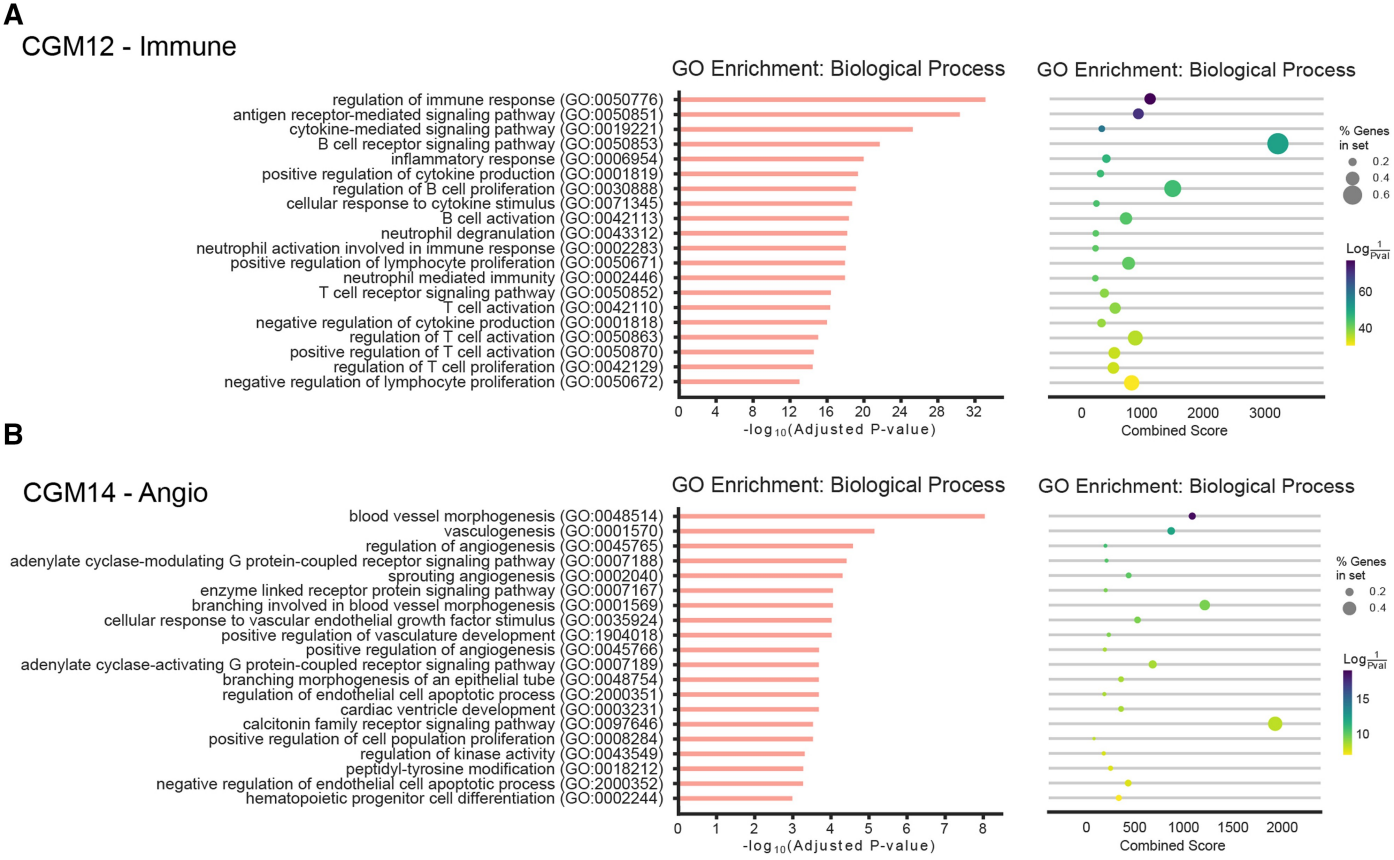
GO Enrichment analysis of IMVigor210 bladder cancer derived CGMs. (A) GO annotation of CGM-12 (Immune). (B) GO annotation of CGM-14 (Angio).

To further validate the biological relevance of identified CGM cores, we performed Gene Ontology (GO) enrichment analysis. For each CGM core, we tested for enrichment across the GO Biological Process database using hypergeometric tests with Benjamini-Hochberg correction for multiple comparisons (adjusted *P* < 0.05). The analysis revealed that CGM cores are significantly enriched for coherent biological functions consistent with their cellular annotations. For example, CGM annotated as immune showed enrichment for GO terms such as “regulation of immune response,” “antigen receptor-mediated signaling pathway,” etc. (Fig. 3, Fig. S1).

Additionally, CGMs were evaluated for their transcriptional coherence using experimentally validated TF–target gene relationships from the ChEA_2022 database (Keenan et al. 2019), as described in the Methods section. We filtered out genes lacking TF annotation in ChEA_2022 and those associated with noise CGMs, retaining 4641 out of 16 834 genes for CGM cores and 6834 genes for CGM modules. The TF knowledge score for CGM cores was 1.312 (*P* < 0.05), and for CGM modules was 1.251 (*P* < 0.05), indicating that CGMs are significantly enriched with gene pairs sharing common transcriptional regulators. In comparison, WGCNA modules yielded a score of 1.041, demonstrating that NestedWGCNA produces more transcriptionally coherent modules.

To further investigate the relation of CGM cores to particular cellular fractions we measured a Pearson correlation of signatures computed based on epithelial and endothelial CGM cores obtained from the IMvigor210 bladder cancer RNA-Seq data (see Supplementary Data 1 and 2) with signature genes characteristic of epithelial and endothelial cell types, respectively, from the PanglaoDB database (Franzén et al. 2019). The resulting correlations were 0.94 and 0.95 correspondingly. These high correlation values suggest that the CGM core signatures reliably capture the transcriptional characteristics of their respective cell types.

### 3.3 Cross-validation of CGM cores on gene cluster plots

**Figure 4:**
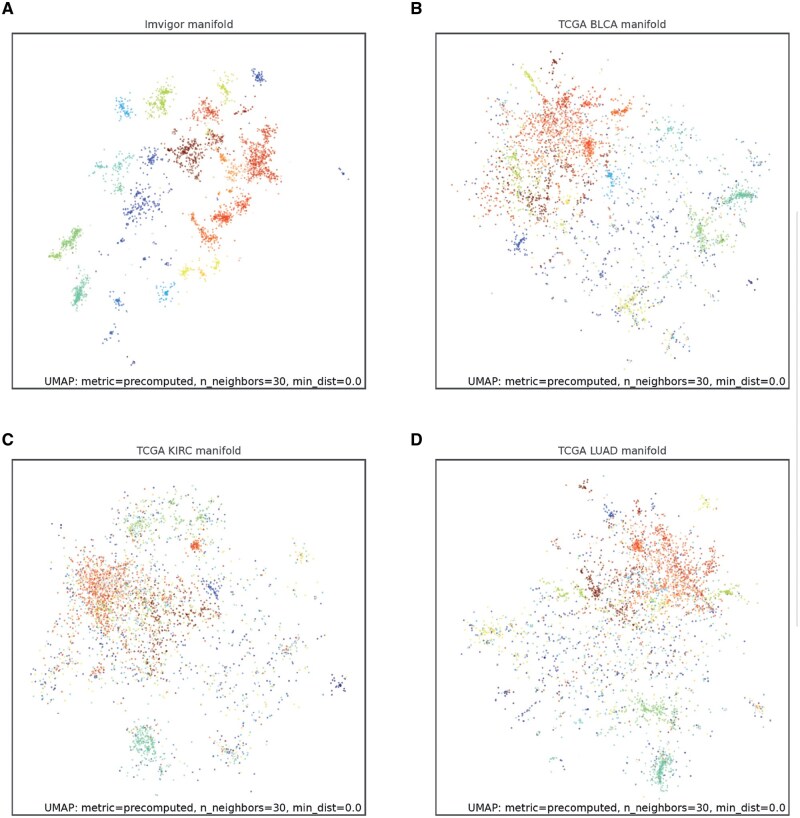
Validation of obtained CGM cores. Congruence of gene clusters across datasets is analyzed. (A–D) CGM cores obtained on IMVigor210 RNA-Seq dataset (bladder cancer) are mapped on the same IMVigor210 (A), TCGA BLCA (bladder cancer, B), TCGA KIRC (renal cell carcinoma, C) and TCGA LUAD (lung adenocarcinoma, D) manifolds. UMAP dimensionality reduction plots are shown.

As an alternative method to assess the algorithm’s robustness, we examined the congruence of CGM core gene sets across datasets. This analysis revealed that CGM cores obtained for one bladder cancer dataset (IMVigor210) generally maintain structural integrity being mapped onto the manifold of another bladder cancer dataset (TCGA BLCA). Mapping IMVigor210-derived CGM cores on lung adenocarcinoma (TCGA LUAD) and clear cell renal cell carcinoma (TCGA KIRC) datasets showed more altered yet relatively structured organization (Fig. 4). Conventional WGCNA-based analysis with default parameters yielded lower structurization and lower gene cluster stability across datasets (Fig. S2).

### 3.4 Validation of CGM cores on scRNA-Seq data

**Figure 5:**
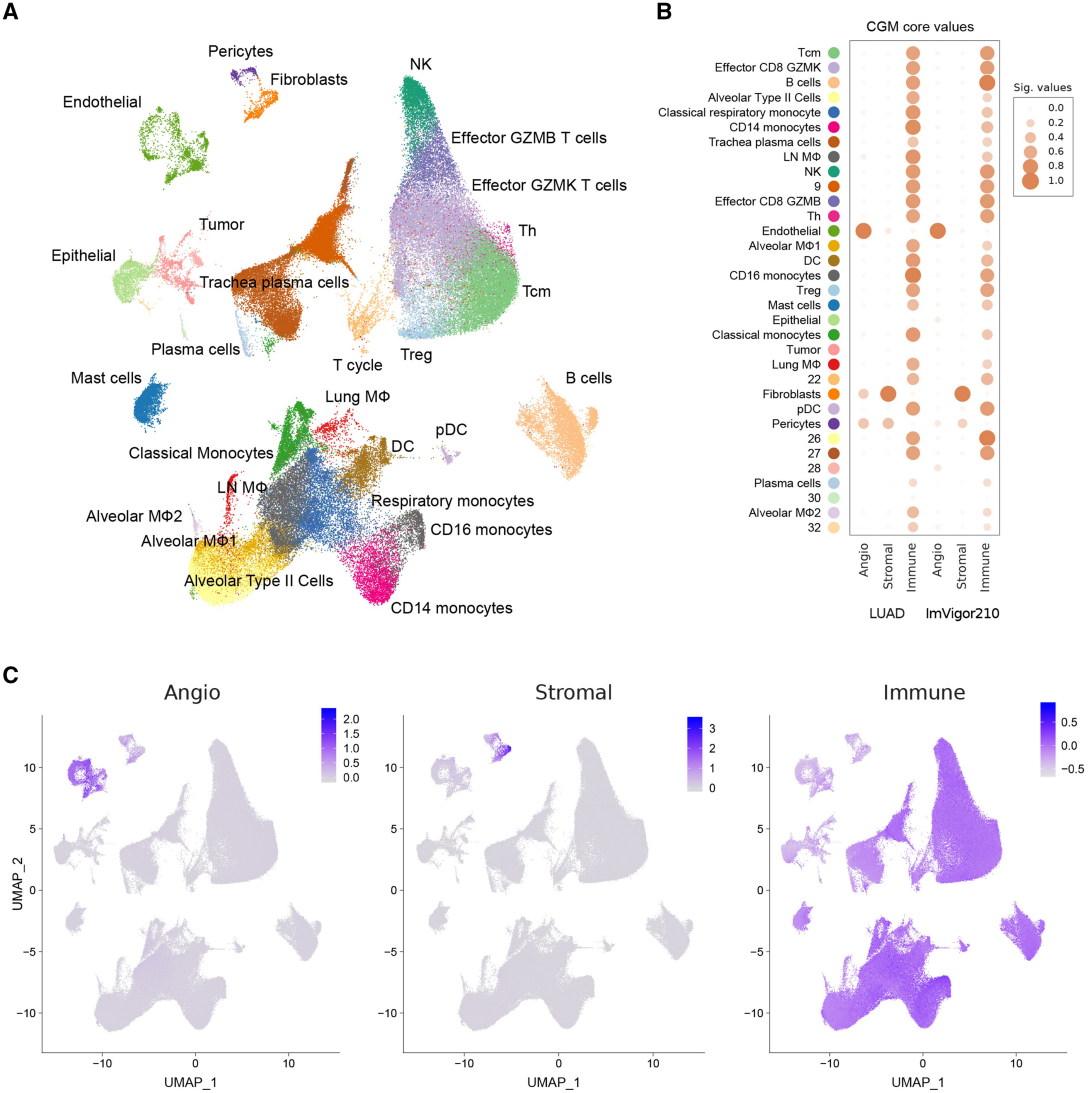
Validation of CGM cores on scRNA-Seq data. (A) Annotated lung adenocarcinoma scRNA-Seq UMAP. (B) Dotplots reflecting the mapping of IMVigor210-derived CGMs on lung adenocarcinoma scRNA-Seq UMAP. Dot size and intensity correspond to average CGM expression level. (C) Mapping of ImVigor210-derived CGMs on lung adenocarcinoma scRNA-Seq UMAP.

Next, we projected CGM cores obtained on IMVigor210 bladder cancer and TCGA LUAD RNA-Seq data onto a scRNA-Seq UMAP obtained for total lung adenocarcinoma cells (Leader et al. 2021, Morabito et al. 2023, Yasumizu et al. 2024) (Fig. 5a). The core CGM genes were used to calculate the corresponding signatures using the Seurat R package (Butler et al. 2018) and the results were applied to a UMAP embedding of scRNA-Seq dataset. Identified CGM cores annotated as *immune*, *stromal* and *angiogenic* were clearly mapped on distinct clusters of scRNA-Seq UMAP manifold (Fig. 5b and c).

### 3.5 Fine-grained modules

Next, we aimed to develop a black box algorithm to identify more compact gene modules that would describe more narrow functional processes (subprograms) or cell subsets. For instance, these modules could encompass distinct programs of angiogenesis within angiogenesis CGM or various types of immune cell behaviour within immune CGM.

To this end, we proceeded to partition the obtained CGMs into smaller gene modules utilizing core decomposition (Fig. 1c). We first normalized all genes of each CGM on the *Core* gene set. The resulting subgraph was next subjected to a second round of co-expression network analysis, yielding this time a larger number of fine-grained modules, FGMs, which disintegrated into smaller clusters in the absence of a primary dominant node (Supplementary Data 4).

FGM represent gene sets that remain correlated even after normalization removes the influence of the major component that initially determined their correlation within the corresponding CGM. This intrinsic correlation between the gene sets is thus independent of the major core process of the CGM, highlighting their self-standing nature. Consequently, we suggest that each FGM reflects smaller subprocesses or specific cell subsets within the larger CGM.

**Figure 6:**
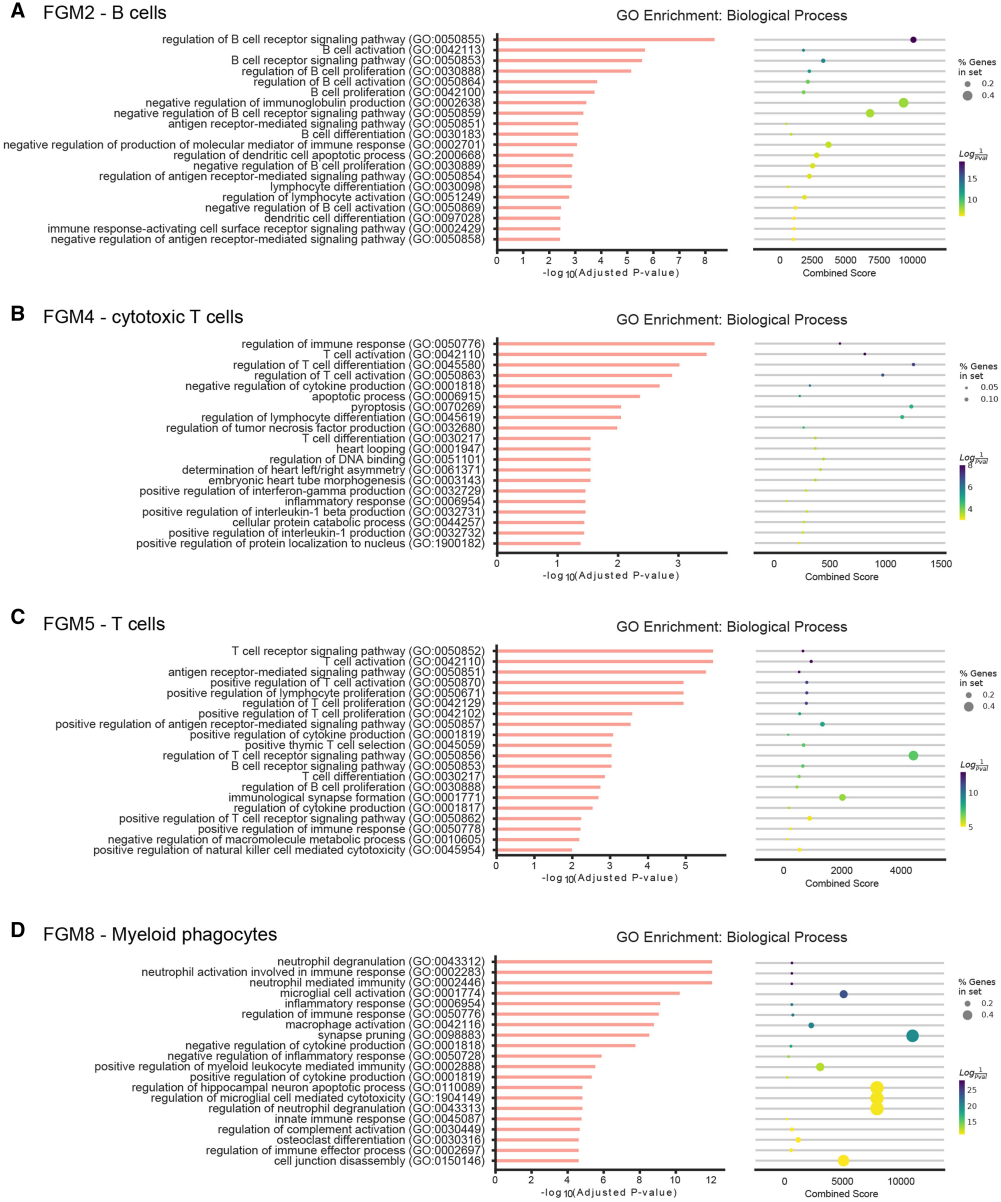
GO Enrichment analysis of IMVigor210 bladder cancer derived FGMs. (A) FGM-2—B cells. (B) GO annotation of FGM-4—Cytotoxic T cells. (C) FGM-5—T cells. (D) FGM-8—Mieloid phagocytes.

To assess the biological relevance of identified FGMs, we performed Gene Ontology (GO) enrichment analysis. Each FGM was tested for enrichment across the GO Biological Process database. The analysis revealed that FGMs are significantly enriched for coherent biological functions consistent with their cellular annotations and functional specialization (Fig. 6). These findings confirm that the identified FGMs capture biologically meaningful and functionally coherent gene sets that correspond to specific cellular processes and regulatory programs within their parent CGMs.

Additionally, we analysed the immune FGMs from the IMvigor210 dataset to assess whether FGMs also capture coherent transcriptional regulatory programmes. Using the TF knowledge score approach with ChEA_2022, 492 of 509 FGM genes were TF-annotated, and the knowledge score reached 1.400 (*P* < 0.05), indicating that genes within the same immune FGM are strongly enriched for sharing a common TF regulator.

### 3.6 Validation of FGMs on scRNA-Seq data

**Figure 7:**
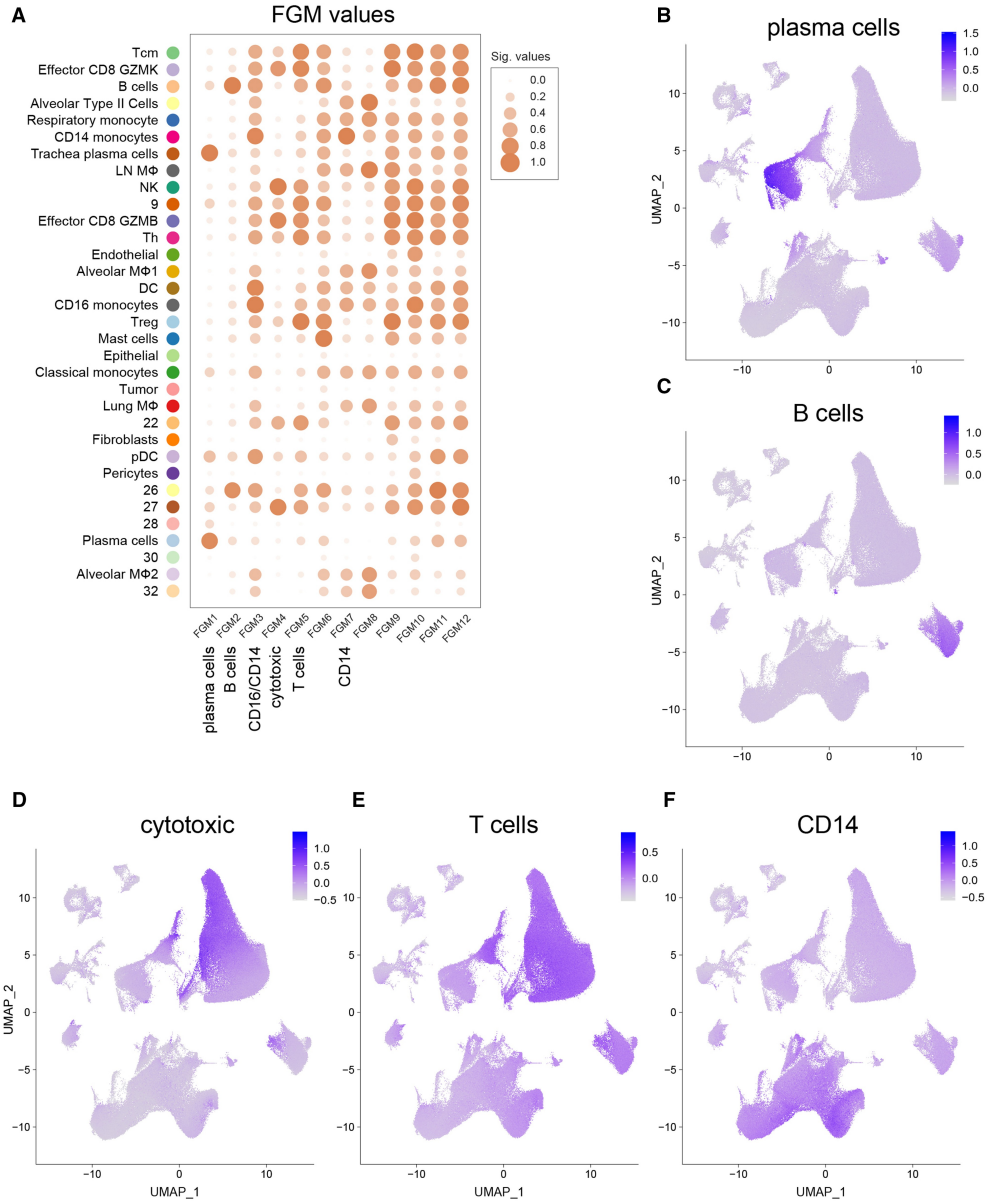
Validation of immune FGMs on scRNA-Seq data. (A) Dotlopts reflecting the mapping of IMVigor210-derived FGMs on lung adenocarcinoma scRNA-Seq UMAP. Dot size and intensity correspond to average FGM expression level. (B–F) Mapping of ImVigor210-derived FGMs on lung adenocarcinoma scRNA-Seq UMAP.

To validate rationality of this approach, we plotted the ImVigor210-obtained FGMs on the lung adenocarcinoma scRNA-Seq UMAP. This allowed us to assess the coherence and consistency of the FGMs assignment within the single-cell landscape. Notably, many FGMs were mapped onto specific locations within the single-cell UMAP, which aligns with the locations overlaid by the CGM cores. In particular, identified immune (Immune CGM-derived) FGMs were associated with distinct cell subsets identified in scRNA-Seq annotation, such as plasma cells, B cells, cytotoxic cells, T cells, CD14 monocytes, CD16 monocytes (Fig. 7). This indicates that our approach indeed allows for the black-box identification of groups of genes responsible for specific functional processes characteristic of particular cellular programs and populations.

**Figure 8:**
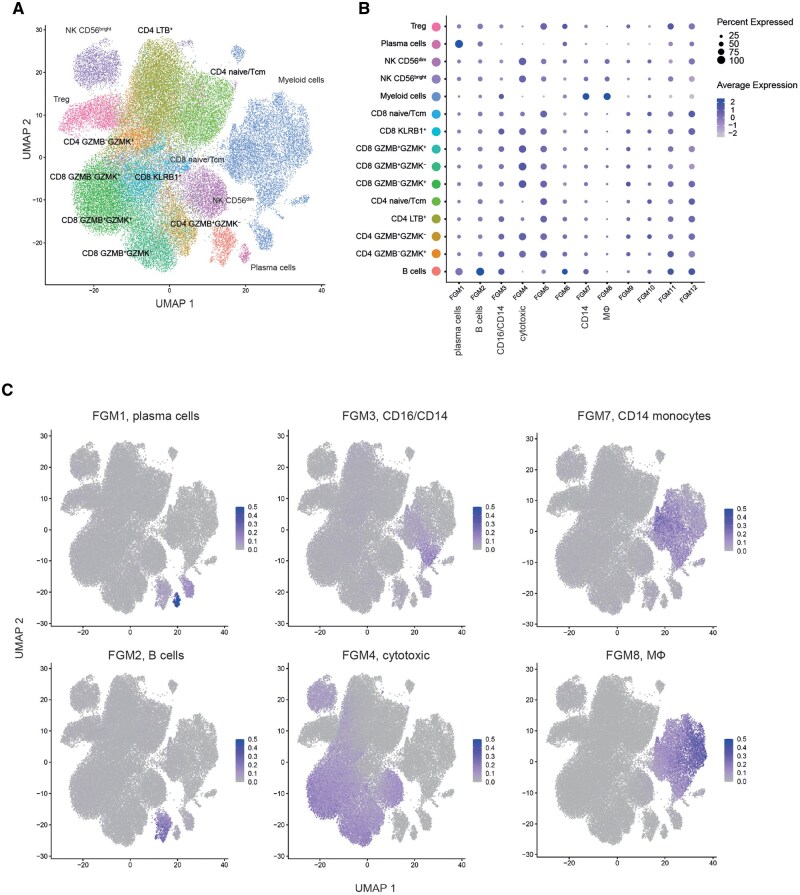
Validation of immune FGMs on atherosclerosis scRNA-Seq data. (A) Annotated atherosclerosis scRNA-Seq UMAP. (B) Dotplots reflecting the mapping of IMVigor210-derived FGMs on atherosclerosis scRNA-Seq UMAP. Dot size and intensity correspond to average FGM expression level. (C) Mapping of ImVigor210-derived FGMs on atherosclerosis scRNA-Seq UMAP.

**Figure 9:**
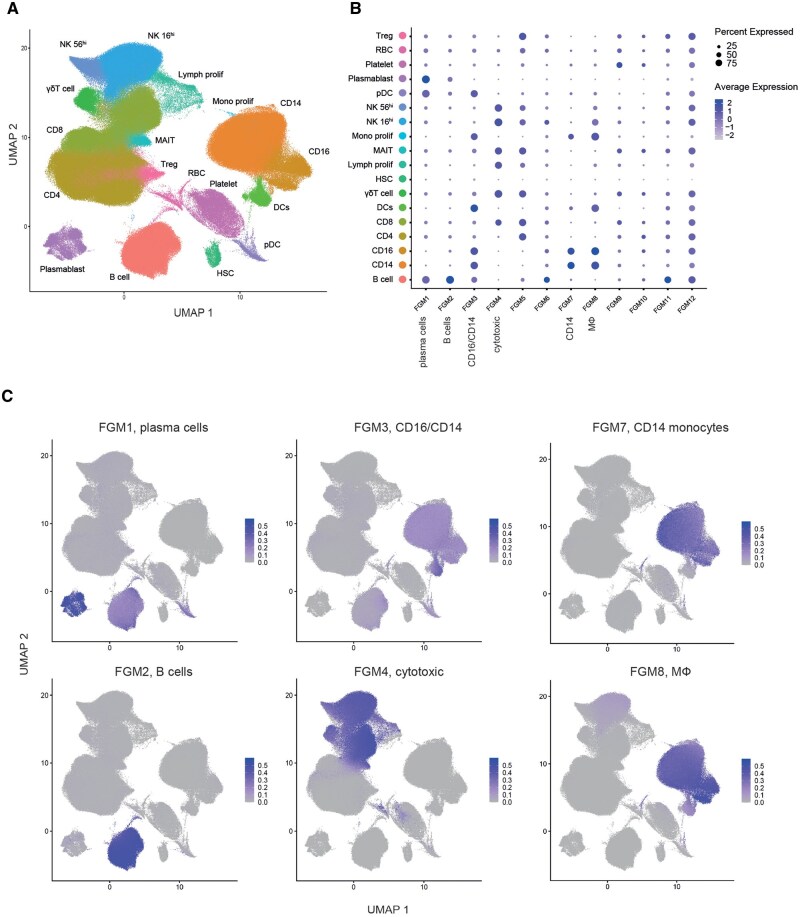
Validation of immune FGMs on PBMC scRNA-Seq data from COVID-19 patients. (A) Annotated PBMC scRNA-Seq UMAP. (B) Dotplots reflecting the mapping of IMVigor210-derived FGMs on PBMC scRNA-Seq UMAP. Dot size and intensity correspond to average FGM expression level. (C) Mapping of ImVigor210-derived FGMs on PBMC scRNA-Seq UMAP.

To further validate the robustness of our approach we applied the same procedure to noncancer scRNA-Seq datasets containing atherosclerosis and COVID-19 patients (Figs. 8 and 9). In both scenarios, ImVigor210-derived FGMs stained nicely the plasma cell, B cell, cytotoxic cell, T cell, CD14 monocytes, CD16 monocytes, and macrophages (MФ) cell subsets, demonstrating the reliability of the determined compact gene signatures and their rational applicability to a wide variety of scRNA-Seq data.

### 3.7 FGMs normalized by CGM core predict immunotherapy response and survival

**Figure 10:**
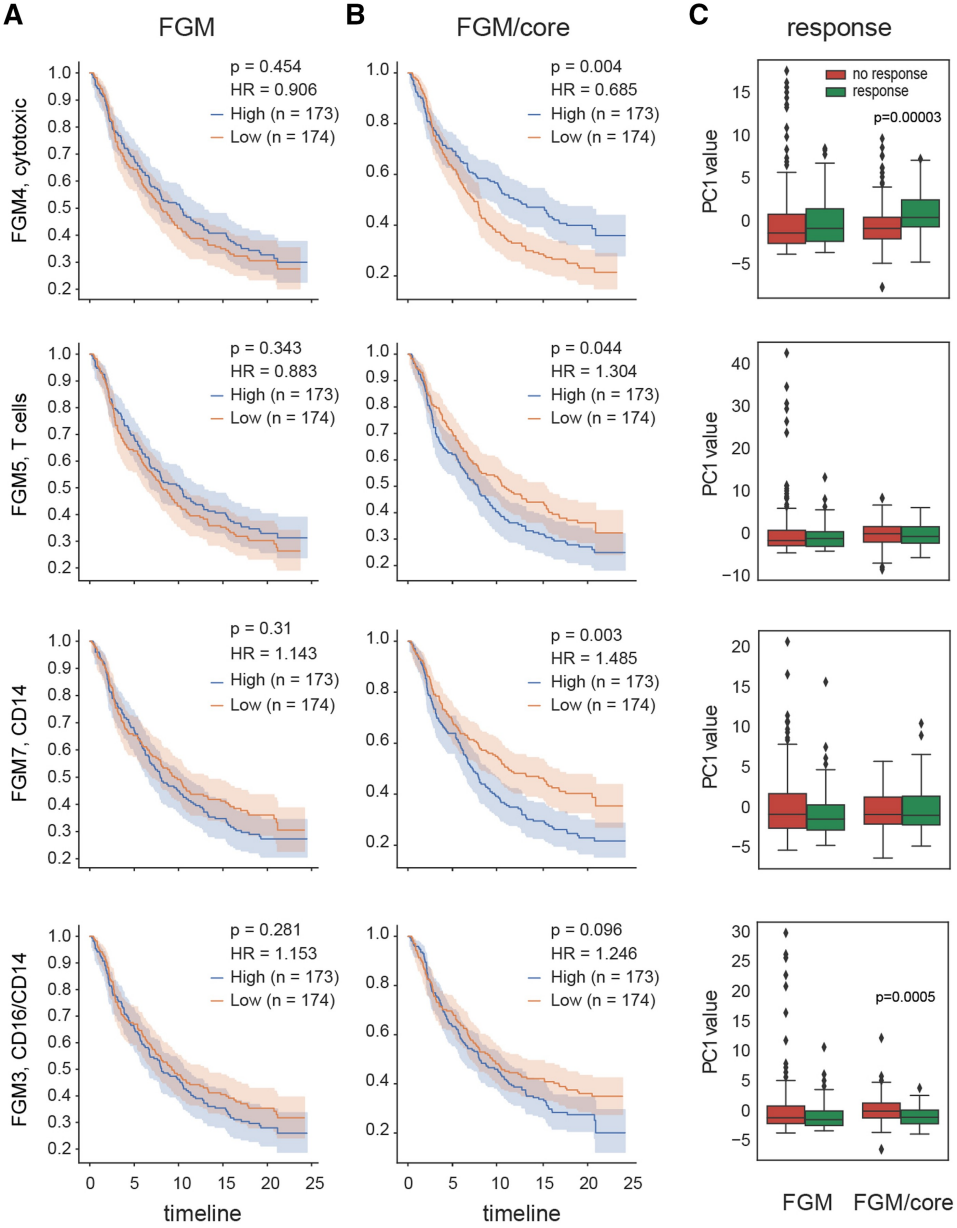
FGM and normalized FGM predicting survival and response. (A and B) Kaplan–Meier curves showing overall survival for IMVigor210 patients with muscle-invasive urothelial carcinoma with high and low FGM expression (A) or FGM expression normalized by CGM core (B). Patient cohorts are divided on the basis of median, with the total number of patients shown in parentheses. (C) Boxplots show associations of high and low FGM and FGM/core expression with response to anti–PD-L1 immunotherapy. Median, bottom quartile, top quartile, and interquartile range are shown.

Being normalized by the corresponding CGM core, FGM4 (annotated as cytotoxic, includes IFNG, SLFN12L, TNFRSF9, CD2, PYHIN1, ITGAL, EOMES, CCR5, CD3G, GZMK, TTC24, PTPN22, NKG7, PDCD1, TBX21, GZMB, ABCD2, GZMA, CD8A genes) exhibited significant association with survival and immunotherapy response. At the same time, FGM4 *per se* demonstrated low predictive power (Fig. 10).

This follows the logic of the “proportion of process within the overall process,” that we recently reported for the Immfocus-like (Teltsh et al. 2015) normalized cytotoxic signature (Mariathasan et al. 2018). Essentially, normalizing FGMs to their respective CGM cores is comparable to normalizing genes associated with specific processes, like cytotoxicity, to a pan-leukocyte marker present on all cytotoxic cells. This parallel underscores the similarity in methodologies, as both aim to capture and quantify distinct immune responses while adjusting for variations in cell composition.

This finding underscores the potential of FGM-based assessments, particularly within immune cell populations, in predicting patient outcomes and guiding personalized immunotherapeutic strategies. The “black-box” identified cytotoxic FGM displays similar associations with survival and response outcomes as the rationally designed cytotoxic signature, both normalized, in a way similar, based on immune cell infiltration. This finding highlights the generalizability of the algorithm’s performance in capturing distinct types of immune responses across different normalization strategies.

Notably, normalized FGM5 (annotated as “CD4 T cells”) was rather associated with negative prognosis (Fig. 10), emphasizing that the quality of tumor-infiltrating T cells is more important than quantity.

It is also remarkable that normalized FGM3 annotated as CD14/CD16 demonstrated a robust negative correlation with treatment response, and normalized FGM7 annotated as CD14 was also inversely associated with survival (Fig. 10). These populations of monocytes were also characterized by low HLA-DR expression on scRNA-Seq analysis of lung adenocarcinoma environment. CD14+HLA-DR^lo/neg^ monocytes are often classified as a type of myeloid-derived suppressor cells (MDSCs), and have been associated with immunosuppression and poor responses to various immunotherapies, including PD-1 and CTLA-4 checkpoint inhibitors, CAR-T cell therapy, and cancer vaccines (Mengos et al. 2019, Ren et al. 2022).

## 4 Discussion

The development of effective cancer treatments remains a significant challenge due to the ability of tumor cells to evade immune surveillance through various tolerance mechanisms. Immunotherapy, which harnesses the immune system to target cancer cells, has revolutionized treatment approaches, but only a fraction of patients responds effectively. This discrepancy, along with development of diverse combinational immunotherapies, underscores the need for reliable biomarkers to predict response and improve patient stratification.

Gene Co-Expression Networks provide valuable insights into gene interactions and collective behavior, with Weighted Gene Co-Expression Network Analysis (WGCNA) being a widely utilized method for identifying gene modules associated with specific biological processes. However, traditional WGCNA has limitations that hinder its robustness and reproducibility. Here we address these limitations by optimizing key aspects of the analysis pipeline. Firstly, we revised the method for constructing the adjacency matrix, moving away from the arbitrary selection of a scale-free transformation parameter. Instead, we employed the Coefficient of Determination, providing a more intuitive measure that enhances the interpretability of the resulting network structure. Additionally, we reevaluated the dissimilarity measure used in module identification, opting for a metric that better preserves the topology of the original data space. By replacing hierarchical clustering with HDBSCAN, coupled with UMAP dimensionality reduction, we achieved more accurate and reproducible module identification, essential for extracting meaningful biological insights.

Next, we introduced a second level of GCN inference, the whole pipeline named Nested-WGCNA (Fig. 1). Conceptual innovation of this approach lies in its ability to distinguish between coarse-grained modules (CGMs) representing major cell types or processes and fine-grained modules (FGMs) capturing more specific cell subtypes and subprocesses. This is achieved by normalization on core genes that removes the major CGM vector, followed by repetition of co-expression network inference. Essentially, each resulting FGM represents a compact group of genes, which remain correlated in the absence of the core CGM vector.

Drawing a social analogy: imagine we need to pick out a group of experts in genomic bioinformatics (FGM) on a campus where 10 000 scientists hang out in the same labs, smoking areas, and cafeterias, all doing what looks like the same stuff. Together, these scientists make up a CGM, which we previously identified as the scientists of this campus in our city’s neighborhood. To find the specific FGM we’re interested in, we need to ignore the main traits of the CGM (like visiting the campus, looking smart, working long hours, wearing lab coats, or chatting with lab coat-wearing folks), and instead focus on something unique that unites the group we’re after (in this case, the habit of spending long hours staring at a Jupyter notebook, real or imaginary).

The strength of Nested WGCNA lies in its ability to identify compact gene groups in a black-box mode, without initial assumptions. For instance, among the FGMs identified in the bulk bladder cancer transcriptomic data, a compact module of genes determining the cytotoxic behavior of immune cells was found. This black-box-identified module fits well into the scRNA-Seq UMAP of lung cancer cells (Fig. 7d) and predicts the response to bladder cancer immunotherapy as successfully as the analytically selected group of cytotoxic genes we described earlier (Dyugay et al. 2022).

Other identified gene groups also reflect specific subprocesses and cell types, including epithelial, endothelial, fibroblasts, as well as various types of myeloid and lymphoid immune cells (Fig. 7). Some of these FGMs, such as those associated with CD14+ monocytes, also have prognostic or predictive power, contributing to overall survival and/or response to anti-PD-L1 immunotherapy (Fig. 8).

Notably, the manifestation of predictive power also requires normalization to core genes, following the logic of ImmFocus (Teltsh et al. 2015). Thus, such normalization is essentially used here twice and independently: the first time to identify a group of genes correlated due to functional commonality in the absence of the impact of the major CGM vector, and the second time to measure the relative strength/representation of the subprocess/cell subset relative to the major process/cell type.

The concept of Nested WGCNA is applicable to the study of various living systems. Black-box identification of FGMs offers an unsupervised zoom-in view into correlating gene networks, allowing for the identification and tracking of specific functional subprocesses via bulk or single cell transcriptomics. By leveraging these modules, we can uncover associations between specific cellular behaviors and treatment outcomes, development or regeneration processes, response to vaccination, subtypes of chronic inflammatory processes, and more.

In cancer immunotherapy, Nested-WGCNA offers a data-driven approach to biomarker discovery with the potential to transform treatment paradigms. The ability to predict treatment response using data-driven features generated by Nested-WGCNA offers a platform to develop personalized approaches that should allow clinicians to optimally tailor combinational immunotherapeutic strategies to individual patients.

Ongoing advancements in single-cell transcriptomics present an opportunity to expand the application of Nested-WGCNA to the analysis of heterogeneous cell populations at unprecedented subprogram resolution. Informative nature of the straightforward cross-cancer projection of FGMs derived on bulk bladder cancer transcriptomic data on lung adenocarcinoma scRNA-Seq (Fig. 7) suggests huge undiscovered potential of the latter direction.

## Supplementary Material

btag167_Supplementary_Data
