# Supplementary material for: Nested co-expression network analysis identifies compact gene clusters in a black box
Source: Bioinformatics. 2026 Apr 3;42(5):btag167. doi: 10.1093/bioinformatics/btag167 (PMC13163169; doi:10.1093/bioinformatics/btag167)
Supplement: btag167_Supplementary_Data [file btag167_supplementary_data.zip › Supplementary information.pdf]

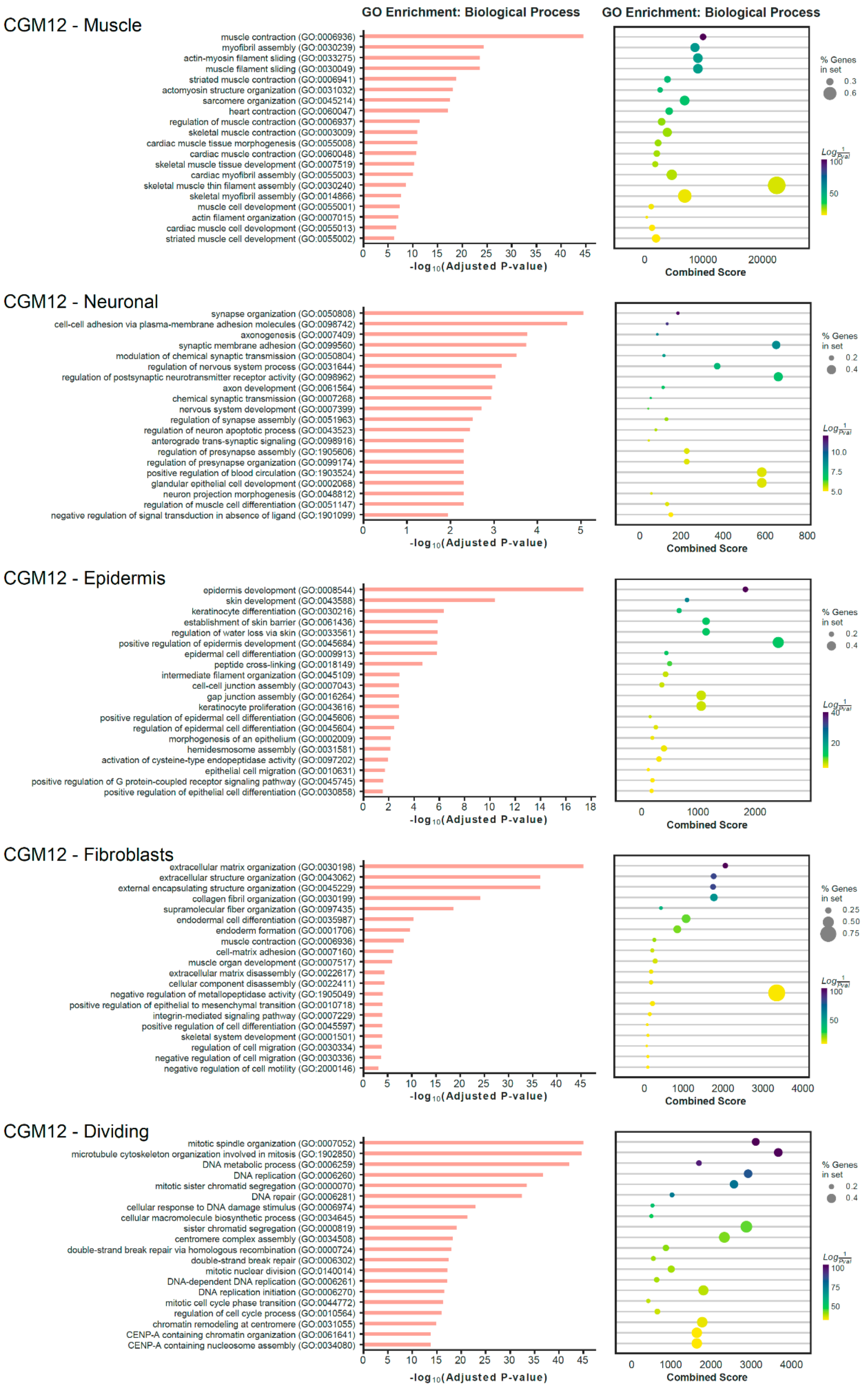

Supplementary Figure. 1. GO Enrichment analysis of IMVigor210 bladder cancer derived CGMs.

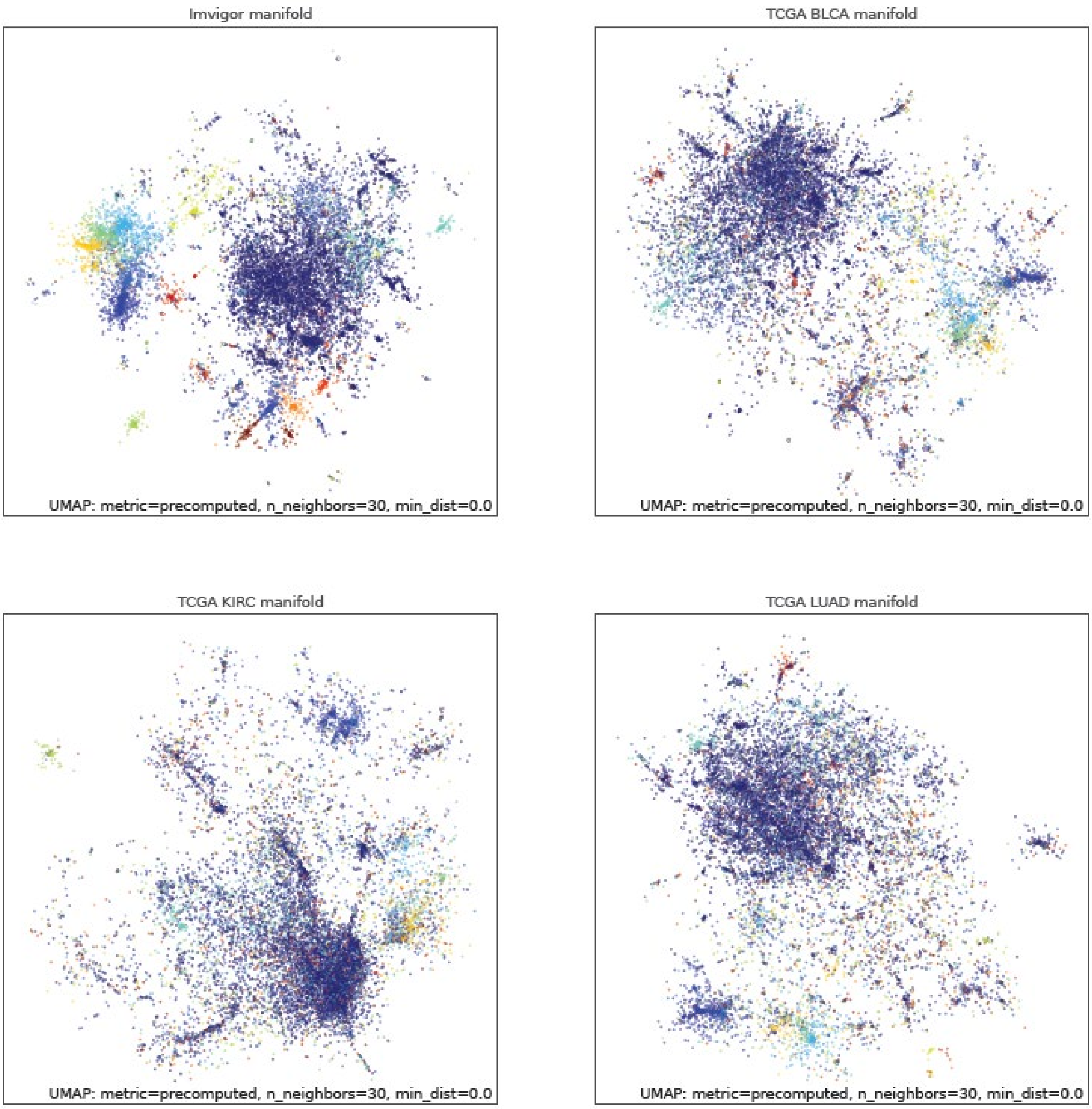

Supplementary Figure 2. **Validation of conventional WGCNA-based gene clusters on UMAP plots**. Congruence of gene clusters across datasets is analyzed. **a-d**. Gene clusters obtained on IMVigor210 RNA-Seq dataset (bladder cancer) are mapped on the same IMVigor210 (a), TCGA BLCA (bladder cancer, b), TCGA KIRC (renal cell carcinoma, c) and TCGA LUAD (lung adenocarcinoma, d) manifolds. UMAP plots dimensionality reduction are shown.

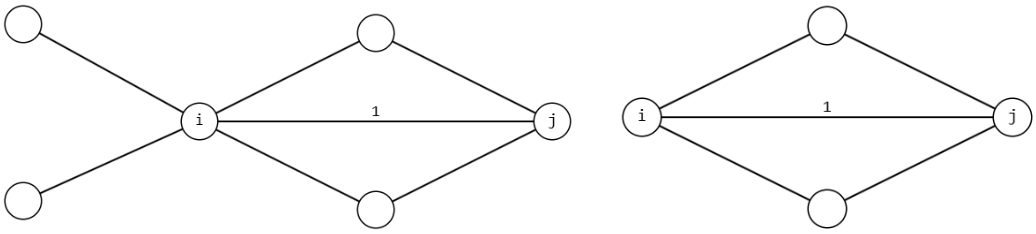

Supplementary Figure 3. **Examples where the WGCNA dissimilarity measure would yield the same result**. Gene i — a hypothetical housekeeping gene in the left part and a part of a closed module on the right.
